# Facilitators and barriers of the implementation of point-of-care devices for cardiometabolic diseases: a scoping review

**DOI:** 10.1186/s12913-023-09419-2

**Published:** 2023-04-28

**Authors:** Janeth Tenorio-Mucha, Patricia Busta-Flores, María Lazo-Porras, Beatrice Vetter, Elvis Safary, Andrew E. Moran, Reena Gupta, Antonio Bernabé-Ortiz

**Affiliations:** 1 CRONICAS Center of Excellence in Chronic Diseases, Universidad Peruana Cayetano Heredia, Av. Armendariz 445 – Miraflores, Lima, Peru; 2 CONEVID - Unidad de Conocimiento y Evidencia, Facultad de Medicina “Alberto Hurtado”, Universidad Peruana Cayetano Heredia, Lima, Peru; 3 FIND, Geneva, Switzerland; 4 Resolve to Save Lives, New York, NY USA; 5 Department of Medicine of Medicine, University of California, San Francisco, USA

**Keywords:** Point-of-care testing, Noncommunicable diseases, Chronic disease, Metabolic syndrome, Implementation science

## Abstract

**Background:**

Point-of-care testing (POCT) devices may facilitate the delivery of rapid and timely results, providing a clinically important advantage in patient management. The challenges and constraints in the implementation process, considering different levels of actors have not been much explored. This scoping review aimed to assess literature pertaining to implementation facilitators and barriers of POCT devices for the diagnosis or monitoring of cardiometabolic diseases.

**Methods:**

A scoping review of the literature was conducted. The inclusion criteria were studies on the inception, planning, or implementation of interventions with POCT devices for the diagnosis or monitoring of cardiometabolic diseases defined as dyslipidemia, cardiovascular diseases, type 2 diabetes, and chronic kidney disease. We searched MEDLINE, Embase, and Global Health databases using the OVID searching engine until May 2022. The Consolidated Framework of Implementation Research (CFIR) was used to classify implementation barriers and facilitators in five constructs. Also, patient, healthcare professional (HCP), and organization level was used.

**Results:**

Twenty studies met the eligibility criteria for data extraction. All studies except two were conducted in high-income countries. Some findings are: 1) Intervention: the most widely recognized facilitator was the quick turnaround time with which results are obtained. 2) Outer setting: at the organizational level, the lack of clear regulatory and accreditation mechanisms has hindered the adoption and sustainability of the use of POCT. 3) Inner setting: for HCP, performing POCT during the consultation was both a facilitator and a barrier in terms of time, personnel, and service delivery. 4) Individuals: the implementation of POCT may generate stress and discomfort in some HCP in terms of training and new responsibilities. 5) Process: for patients, it is highly appreciated that obtaining the sample was simple and more comfortable if venipuncture was not used.

**Conclusion:**

This scoping review has described the facilitators and barriers of implementing a POCT device for cardiometabolic conditions using the CFIR. The information can be used to design better strategies to implement these devices and benefit more populations that have low access to cardiometabolic tests.

**Supplementary Information:**

The online version contains supplementary material available at 10.1186/s12913-023-09419-2.

## Background

Point-of-care testing (POCT) devices can deliver rapid and reliable results, giving a clinically important advantage in patient management by allowing healthcare professionals to make timely decisions regarding diagnoses and treatments [1]. A systematic review of qualitative studies from 2013, reported that primary care clinicians believed POCT improved diagnostic certainty, targeting of treatment, self-management of chronic conditions, and clinician-patient communication and relationships [2].

POCT is as useful as laboratory testing to identify risk factors, diagnosis and continuous monitoring of cardiometabolic diseases such as hypertension, diabetes, or congestive heart failure, among others [3, 4]. POCT devices can test single parameters such as haemoglobin A1c [5], glucose [6], lipid profile [6], or serum creatinine [7] one at a time, or multiple haematological parameters at once with multiplex cartridges [8, 9]. Their use in limited-resource settings is attractive because they grant results within minutes, are easy-to-use, and are small in size, which makes them easy to place in clinics with limited space or—for some devices – being portable. In addition, they are frequently less costly for the patient as they eliminate the need of travelling to another facility for testing or for sample transport to a laboratory [10].

Implementation of POCT in low-and-middle income countries (LMICs) has been assessed mostly for infectious diseases, such as malaria, Human Immunodeficiency Virus (HIV), Human Papillomavirus (HPV), dengue, Ebola and Zika viruses, and tuberculosis [11]. The increasing prevalence and morbidity caused by sexually transmitted infections has created interest in the development and implementation of POCT for these diseases, including the development of criteria by the World Health Organization (WHO) [12], which establishes that the ideal POCT should be Affordable, Sensitive, Specific, User-friendly, Rapid and Robust, Equipment-free and Delivered to end users, referred as ASSURED.

The WHO Package of Essential Noncommunicable Disease Interventions for primary health care in low resource settings [13], includes the use of POCT devices as acceptable quality interventions that could aid in the detection and management of cardiometabolic risk. However, the utilization of POCT for noncommunicable diseases is in an early stage in these settings in spite of the increasing morbidity and mortality of cardiometabolic diseases. A better understanding of the existing barriers and facilitators in the utilization and/or implementation of POCT devices for cardiometabolic diseases which mostly considers experiences from high-income settings [14] may inform strategies that need to be taken into account in low-resources settings when implementing POCT. Moreover, the challenges and constraints in the implementation process considering different actors may help to identify action plans at different levels. Hence, this scoping review aimed to assess literature pertaining to implementation barriers and facilitators of POCT devices for the diagnosis or monitoring of cardiometabolic diseases.

## Methods

### Purpose of the scoping review

We conducted a scoping review, as its methodology is suitable to map the existing literature and identify research gaps in the implementation process of POCT devices for cardiometabolic diseases in comparison with POCT for infectious diseases, as well as differences by setting and country income. We adopted the PRISMA Extension for Scoping Reviews (PRISMA- ScR) [15] to guide this review (See checklist in Supplementary table 1). No protocol registration has been done.

### Eligibility criteria

#### Inclusion criteria

Studies were eligible if they involved the inception, planification or implementation of interventions with POCT devices for the diagnosis or monitoring of cardiometabolic diseases through observational, or experimental designs, either with quantitative and/or qualitative methods. Studies informing variables related to effectiveness, efficacy or implementation strategies, as well as hybrid effectiveness-implementation outcomes were included. Thus, implementation outcomes may include acceptability, reach, adoption, fidelity, implementation cost and sustainability.

Included studies had to clearly mention they studied some device, it could be an apparatus, machine, or instrument intended for the determination of some biochemical parameter. This review focused on POCT devices for the diagnosis or monitoring of cardiometabolic diseases defined as dyslipidemia, cardiovascular diseases (CVD), type 2 diabetes (T2D), and chronic kidney disease (CKD). Initially, the study selection was not restricted by health conditions because we were concerned about the lack of reports about facilitators and barriers with an emphasis on cardiometabolic diseases. We found studies on infectious diseases, ultrasound, pregnancy and neonatal, and cardiometabolic diseases. Hence, following our main objective, this article only synthesizes information on cardiometabolic conditions.

#### Exclusion criteria

We excluded studies that reported algorithms for diagnoses such as risk calculators or risk scores and/or rapid diagnostic tests.

### Sources of evidence

We conducted a literature search on MEDLINE, Embase, and Global Health databases using the OVID searching engine. We have used a combination of terms related to primary care, health care, implementation, point-of-care technology, and facilitators and barriers. Details of the search strategy can be found in the Supplementary table 2. The search strategy was not restricted by date, language, or study design to capture the full range of literature related to our review question.

The results from the search were compiled by one author (ABO) who removed duplicates in EndNote® and after that uploaded results to Rayyan, an open- source software that allows for collaboration when screening and selecting studies for systematic and scoping reviews. The search was performed on May 18, 2022.

### Selection process

The articles were selected by three reviewers (ABO, JTM, and PBF) who independently selected potential articles of interest based on the title and abstracts. At this stage, differences in reviewers’ selection were solved by majority. Subsequently, the same three reviewers screened full texts. Disagreements were discussed by the review team until consensus was reached.

### Data extraction

Data was extracted by two reviewers independently (JTM and PBF) using an Excel Sheet (Microsoft Corp). Verification of the extracted data and resolution of any discrepancies was performed by a third reviewer (ABO). Data extracted included: bibliographic details (first author, year of publication, and country), objective, study design, setting, characteristics of the POC device, characteristics of the intervention/implementation, and the findings related to the main objective of the study. For facilitators and barriers of the implementation of the POCT devices, statements were extracted textually.

### Data synthesis

We used the Consolidated Framework for Implementation Research (CFIR) as well as the level of influence. This method was previously used by Sung et al. [16] to explore the implementation determinants of health information technology for non-communicable diseases. The CFIR framework [17] has been extensively used in implementation research. It is composed of five constructs: 1) Intervention: the innovation being used or implemented, 2) Outer setting: the environment or context where the inner setting exists, 3) Inner setting: the setting(s) where the innovation takes place, 4) Individuals: roles and characteristics of individuals, and 5) Process: the activities and strategies used. As CFIR is limited in identifying specific stakeholders, we categorized the outer setting, inner setting and process constructs in three levels of influence: 1) patients, 2) health care professionals (HCP), and 3) organizational. The Individual’s construct was only categorized in two levels: patient and HCP. However, the Intervention construct was not categorized, as it is specific to the characteristics of the POCT device implemented.

## Results

### Characteristics of studies included

**Fig. 1:**
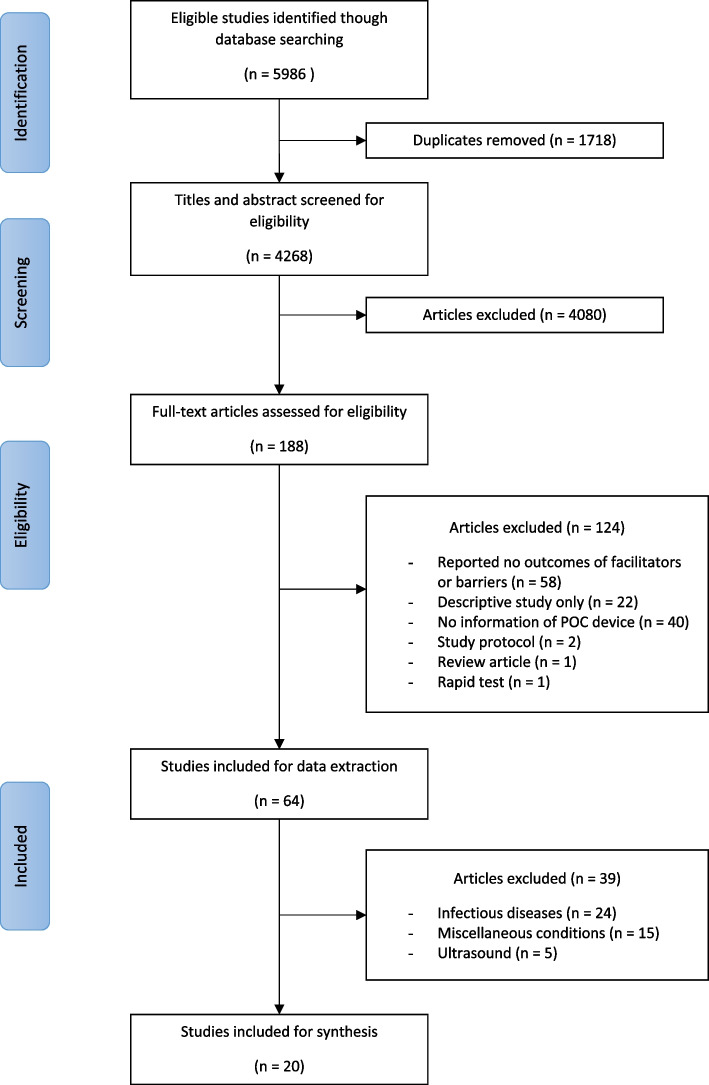
Study selection process

Twenty studies met eligibility criteria for data extraction and were organized them according to the thematic classification. Figure 1 describes the selection process. The selected studies comprised of: six cross-sectional [18–23], six implementation studies [24–29], two cohorts [30, 31], two randomized trials [32, 33], one quasi-experimental [34], one case study [35], one audit [36], and one pilot study [37]. Regarding the methods of the study, 10 were quantitative only [18–21, 26, 30, 31, 33, 36, 37], five qualitative only [22–24, 27, 35] and five had a mixed-method approach [25, 28, 29, 32, 34]. Included studies were published between 2006 and 2022, and in English language. All studies except two [20, 34] were conducted in high income countries (HIC). Twelve studies were performed in primary health care [18, 19, 23–26, 28, 31–34, 37], six hospital-based [21, 22, 27, 29, 30, 36], one during a disaster crisis [20] and one in the British national health service [35]. Among the reported POCT devices, five were used for T2D [19, 22, 27, 30, 34], seven for cardiovascular diseases [21, 29, 31, 32, 35–37], and eight address more than one condition [18, 20, 23–26, 28, 33].

High levels of satisfaction with the use of POCT have been reported among patients [30, 33], HCP [25, 27], and decision-makers in healthcare centers [25]. POCT had a good test performance with high sensitivity and specificity compared to laboratory testing [19, 31]. The good perception of the usability of the device improved patient’s experience has driven successful adoption [24]. In addition, POCT are a good option in emergencies, disasters or humanitarian crises [20].

After the implementation of POCT, the findings on improvement in clinical outcomes are conflicting. On the positive side, there was a significant improvement in HbA1c using POCT compared to laboratory testing [30], a reduction in length of stay [36], and a reduction of hospital referral of low risk patients [37]. On the contrary, others reported no clinical improvement in glycemic control [34], no usefulness completing CVD risk assessment [29, 32], and no widespread adoption or implementation [28, 29, 35]. Regarding financial aspects, it was reported that POCT decreased the total cost per patient admission, but reagent cost increased the laboratory charges [36]. More details can be found in Supplementary table 3.

### Construct 1: intervention characteristics

The most widely recognized facilitator is the quick turnaround time with which results are obtained, while the greatest barrier is the cost to the healthcare facility, either because of implementation expenses or the purchase of reagents and consumables. Implementation costs imply the allocation of resources for additional staff training and their time, device maintenance, and data management. Accuracy and data quality of POCT devices was recognized as facilitator, accordingly, the uncertainty about these characteristics were perceived as barriers (though in one study testing inaccuracies were attributed to patient condition and/or the setting infrastructure rather than the device [27]). Simplicity in design and user-friendly protocols facilitated adoption of the device [24, 31, 34]. For devices that assessed one parameter only, HCP expressed a desire to measure other parameters with the same device [34]. Detailed facilitators and barriers are shown in Table 1.

**Table 1:** Construct 1 (intervention characteristics): Facilitators and barriers in the implementation of POCT for cardiometabolic diseases

Facilitators	Barriers
- Turnaround time for POCT was shorter than laboratory testing [21, 24, 31, 35] - Simplicity influences ongoing clinical engagement by improving it [24, 34] - Convenience in obtaining rapid results [26, 28, 34, 37] - Positive perceptions regarding POCT accuracy, data quality, and quick validation process [21, 25, 27] - POCT is perceived as more convenient than laboratory service [25] - When POCT device was familiar to HCP, there was little questioning of devices’ functioning [35] - Staff satisfaction when no calibration is needed [31] - Integration [27, 37] or desire of integration of the POC testing post trial [27, 32]	- Implementation costs caused health services costs to increase [21, 22, 35, 36] - High cost of consumables or reagents [32] - Not detailed protocols on how to use the device or identify abnormal parameters (HCP faced difficulties interpreting the results) [28] - Perception that laboratories have better hygiene than POCT space [18, 33] - Difficulties with handling and storage conditions that may damage packing, cartridge, or test strips [18, 24, 28] - Device design does not always correspond to customer wishes [22] - There are parts of the device difficult to work with [22] - Perception of low data quality and fear of problems due to false negatives results [21] - Complex device set-up procedures [27] - Limited user-friendliness for primary care [31] - Consumables have short shelf life [35] - Perception that there was no significantly improvement in health outcomes nor in the number of patients who receive care [34]

### Construct 2: outer setting

At patient level, we did not identify any statements as barriers. Among the facilitators, obtaining results on the spot and in a single consultation is the main reason for patient’ satisfaction. At the HCP level, POCT devices are useful for quick response and early decision-making. Diagnostics and treatment can be provided in a single consultation, avoiding long waiting times to pick up the results or to have a new consultation. In some cases, it even contributes to multidisciplinary decision-making [28] or assisted the management of patient with complex comorbidities [23]. The lack of clear regulation and the management of borderline results are the main concerns for HCP. At the organizational level, the lack of clear regulatory and accreditation mechanisms has hindered the adoption and sustainability of the use of POCT. It is perceived as a barrier that the use of POCT is not prioritized by decision-makers [35]. The adoption and/or implementation of new POCT must be supported by clinical evidence [35]. The implementation process should involve multiple stakeholders both internal [22] and external [23]. Detailed facilitators and barriers are shown in Table 2.

**Table 2:** Construct 2 (outer setting): Facilitators and barriers in the implementation of POCT for cardiometabolic diseases

**A. Patient level**	
**Facilitators**	**Barriers**
- Convenient, as saving patient from travel to a facility [23, 28, 30, 33] or to an outside laboratory [33] - Patients were financially satisfied when they did not have to pay more for their medical care [30] - Increase satisfaction with the pathology services provision [25] - Reduced number of patient visits [26, 34]	None mentioned
**B. Health care professional level**	
**Facilitators**	**Barriers**
- Biomedical scientist perceived their technical capabilities will be better valued [21] - POCT assisted the management of acutely ill patients [25, 29] or with complex comorbidities [23] - POCT provide access to information for early decision-making [23, 28]	- Potential medico-legal risk of obtaining and managing abnormal or borderline results [22, 28] - Concerns about regulation and accreditation of POCT [21]
**C. Organizational level**	
**Facilitators**	**Barriers**
- Integration of all relevant areas and organizational units (such as laboratory, information technology, nursing and administration) enables the adoption and sustainable use of POC testing [22] - Set a realistic plan of the implementation with all stakeholders including all driving and all obstructive players facilitates POC adoption [22] - Open communication and regular forwarding of information with the leading areas [22] - Availability of supplies and technologies guaranteed by systems are needed to sustain the use of POCT [23] - Making the POCT device available as part of the team role (multidisciplinary decision-making) [28] - Direct marketing to decision makers encourages a cultural shift toward POCT [35] - Facilitate communication within the team and between them and externals [23]	- Accreditation and regulation problems impede clinics to order supplies [22, 28] - Shortage of cartridges in the market [19] - Unclear requirement profile or statement regarding necessary changes in information technology infrastructure on the part of the manufacturer [22] - Not aligned with local National Health Services interest [35] - Core groups like IT often not included since the beginning in preparation (by hospital) for a decision [22] - The adoption of POCT is not a priority [35] - There was also concern (primarily from commissioners) that once the test was easily available, it would be used for other indications leading to an increase in referrals and therefore costs [35] - POCT must be overseen by the pathology laboratory [21]

### Construct 3: inner setting

The promptness in receiving the results and the explanations provided by the health personnel was highly valued by patients. For HCP, performing POCT during consultation was recognized by some as a facilitator, but by others as a barrier. As a facilitator, since it paves the way for counselling on awareness of the health condition, lifestyle, or adherence to medication. As a barrier because it needs the allocation of extra staff time, or it may disrupt the consultation flow. For clinical management, POCT could assist in early detection [24] and action to manage risk factors [32–34]. At the organizational level, the main barriers reported were infrastructure issues (e.g. space to place the device [32], storage conditions [18], or transportation [24]), high personal turnover, and workload. Detailed facilitators and barriers are shown in Table 3.

**Table 3:** Construct 3 (inner setting): Facilitators and barriers in the implementation of POCT for cardiometabolic diseases

**A. Patient level**	
**Facilitators**	**Barriers**
- Patients feel the quality of services was visibly improving [34] - Satisfaction with the promptness with which patients were assessed [37] - Patients satisfaction with the explanation about the test and procedures [30, 37] - Satisfaction to be able to have immediate feedback from the clinician [26, 30, 34]	None mentioned
**B. Health care professional level**	
**Facilitators**	**Barriers**
- Decrease exposure to other infectious diseases (i.e., COVID-19) [27] - HCP positive perception towards overall time saving [27] - Receiving the results immediately drove discussion about the meaning of the CVD score [32], facilitated disease management [30, 33], and helped to motivate lifestyle change [34] or compliance with taking medication [25] - POCT was able to improve clinicians’ understanding of their patient’s physical health and can help them to communicate results [24] - POCT increase the possibilities to provide physical health checks [24] - Clinicians reflected on the advantages of early identification of metabolic pathology, especially when POCT hastened detection compared to traditional care pathways [24]	- Concerns about the impact of testing on the pressures on the service, for example generating work and uncertainty in response to large numbers of indiscriminate tests done for uncertain indications [23] - Need to allocate extra staff time [32] - Disruption of consultation flow sometimes led to clinicians abandoning the use of the device [24] - Associated protocols and training material crowded nurses’ workstations [27] - Concerns about lack of demand for POCT or duplication of laboratory test [21] - No difference in the completion of CVD risk assessment [32] - The test would not add extra value to clinical assessment or management [29]
**C. Organizational level**	
**Facilitators**	**Barriers**
- Sharing experiences about the test with colleagues could be a valuable way to learn about and support usage of the equipment [28] - POCT may support the need of a hospital admission for an abnormal result [23]	- The need of a space for the POC device and for performing the test [32] - High personnel fluctuation on the wards generates low motivation to become familiar with the device [22] - Integration of the innovation into the existing system is made difficult [22] - The device requires space, time and staff to operate it and to interpret the results [35] - Having only one device was restrictive, as only one clinician could use it at a time and in one location [24]

### Construct 4: characteristics of individuals

POCT strengthened the relationship between the patient and HCP [30]. Having the results during one single consultation can result in higher patient trust of the diagnosis and provides an opportunity for the HCP to provide education on the health condition. Nevertheless, the implementation of POCT may generate stress and discomfort in some HCP in terms of training [24], new responsibilities [27, 29], and approaches to manage risk and clinical uncertainty [28]. Detailed facilitators and barriers are shown in Table 4.

**Table 4:** Construct 4 (characteristics of individuals): Facilitators and barriers in the implementation of POCT for cardiometabolic diseases

**A. Patient level**	
**Facilitators**	**Barriers**
- Patients appreciated results to allow reassurance or to inform further care decision [28] - POCT increased patient’s confidence and offered objective validation for clinical assessment and decision making [23]	None mentioned
**B. Health care professional level**	
**Facilitators**	**Barriers**
- Some HCP relish the opportunity to learn a new skill and develop professionally [24] - HCP enjoy responsibility for testing [25] - HCP felt their skill level increased autonomy and control POC testing gave them [24]	- Laboratory and clinical staff were resistant to delegate testing responsibilities to nurses or pharmacist who work in out-of-facility [35] - HCP reported feeling time-pressured with their workload [27, 29, 32] - Rejection/lack of motivation on the wards towards something “new” [22] - Insufficient laboratory knowledge by users leads to lack of understanding for the importance of quality control of the devices [22] - Some HCP felt that the results’ interpretation may vary depending on staff training and degree of experience [28] - Fear of technical innovations [22] - Some HCP are affected by the anxiety of learning a new skill and fitting it into the workload [24] - Some HCP have doubts if the device’s introduction was worthwhile for the clinical practice and health indicators [24] - Perception that the immediate access to blood test would not change management or would not add to existing clinical assessment [28]

### Construct 5: process

For patients, it is highly appreciated that obtaining the sample was simple and more comfortable if venipuncture was not used. HCP considered that protocols must be simple [27] and everyone should follow when using the POCT, especially those for quality control [18]. Furthermore, it is recommended that the first training must be well done and must be accompanied by continuous reinforcement. A way to facilitate the implementation of POCT was to have support systems which provide guidance and supervision [23]. There was also a need for proper logistic coordination and process standardization. Detailed facilitators and barriers are shown in Table 5.

**Table 5:** Construct 5 (process): facilitators and barriers in the implementation of POCT for cardiometabolic diseases

**A. Patient level**	
**Facilitators**	**Barriers**
- Patient satisfaction when the sample is obtained at first try [26] - From a patient perspective, the finger prick was better than venipuncture [30, 32–34]	- For some patients, POCT might result in unnecessary duplication of work [24] - For devices that needs venous blood, there are difficulties obtaining the sample [28]
**B. Health care professional level**	
**Facilitators**	**Barriers**
- Staff trained by other clinic staff performed as well as people with formal accredited training [19] - POCT could serve for education and reassure patients [35]	- Few users read the test procedure written specifically for the practice or the instructions for use before using a POCT [18] - Laborious calibration process and long analyzer warm-up [31] - The protocol was excessively lengthy and nurses expressed a desire for simplification [27] - The need of performing mathematical calculations was a barrier for some nurses [27] - Qualitative tests are subjective and judged moderately difficult compared to the quantitative test [31] - Non-adherence to POCT procedures [18, 29]
**C. Organizational level**	
**Facilitators**	**Barriers**
- Manuals and posters have were preferred than DVD as instructive and appropriate for training [25] - Training should consider the need of the staff, how they understand and interpret results as well as how to use the device [28] - Have system level support for the effective implementation [23]	- Lack of introduction of training [22] - Time delay between training and actual introduction and hence also adaptation of the innovation [22] - Refresher courses are hardly ever organized, even when the test or the instructions for use are modified [18, 22] - Lack of responsibility for new devices on the wards [22] - Lack of standard operating procedures [22] - Need for improved results registries and simplified administrative protocols [27] - Logistic often exclusively focus on proof of economic benefit, while underestimating importance of qualitative, risk-reducing aspects [22] - The most frequent issue was the bulkiness of the device and the subsequent difficulties of transporting [24] - Additional delays through extensive coordination process [22]

## Discussion

This review has revealed commonalities as well as particularities in the implementation of POCT for different cardiometabolic parameters, settings, and target populations. The findings were organized in constructs and level of influence allowing a comprehensive understanding of barriers and facilitators reported in the literature. Furthermore, we have confirmed a substantial gap in the information of implementation of POCT devices for cardiometabolic diseases in LMICs: Only two studies identified were performed in these settings: one in an urban area in South Africa [34] and another one including Thailand when affected by Hurricane Katrina [20].

There is more evidence about POCT utilization on infectious diseases in high income settings as well as in LIMCs which report similar challenges as our review, especially those related to the constructs of inner and outer setting, and process. Among them, we can highlight: the lack of communication between different stakeholders in the healthcare system hinder the adoption and scaling-up of POCT usage [38, 39], the increase workload for HCP discouraging them to use new technologies [39, 40], and the differentiated background among users (e.g. medical/non-medical or specialized/non-specialized) plus the absence of continuum training cause lack of confidence in the POCT and the results [41, 42]. Nevertheless, common facilitators were identified, such as: HCP enthusiasm to obtain same-day results [43], reduce re-consultations [14, 40] and save patient’ travel expenses [44], the test influence patient-HCP interaction [45, 46], and the non-requirement of specialized skills to operate the devices [47]. Future implementation of POCT for both infectious diseases and NCDs should learn from the facilitators and barriers previously identified when designing the components of the intervention in order to find ways to take advantage of facilitators and/or overcome barriers.

This review identified that in many cases the device design does not fit customer wishes: some specific problems reported are the lack of space to place POCT devices and for performing the test, difficulties in transport, and complex set-up procedures. These can be more problematic in LMICs where healthcare systems are often ill-equipped: there are areas with lack of access to reliable power supply, and some facilities may be located at extreme weather conditions [48]. For example, during summer in Haiti, the staff was forced to perform trial and error tests with the POCT device in cloth packing surrounded with ice to avoid the device reading error codes when ambient temperatures are out of range [3]. Although, it is mentioned they periodically verify the device readings with laboratory confirmed values [3], there is uncertainty about the precision of the results. Interdisciplinary research and transcontinental collaboration is needed to develop not only sophisticated devices but also those which can adapt to the environment and context of limited-constraint settings [49]. Furthermore, POCT alone is not enough to improve patient diagnosis and disease control, it must be accompanied by changes in processes at clinical and organizational levels with full recognition and adherence to evidence-based practice [50].

An important concern related to the introduction of POCT is the implementation costs [21, 22, 35, 36] and the procurement of reagents and consumables [32] which in consequence may increase the cost of health services utilization. A review about economic evidence on POCT [51] recognized that cost-effectiveness evaluation should assess cost not only on the value of test but also in the impact on health outcomes, process of care, and on resource utilization. Currently, the literature about cost-effectiveness about POCT is weak, just a few studies reported savings in the utilization of resources such as hospital admission or length of stay [52] or cost minimization in delivery of care at primary level [53]. Considering cardiometabolic diseases require ongoing clinical monitoring to assess control and thus prevention from suffering complications, a specific cost-effectiveness analysis is required accounting health, process, and resources outcomes.

We recognize some limitations in this review such as the lack of assessment of the quality of the studies included. Also, the analysis of facilitators and barriers were based on reports from previous studies and some findings can be subjective. We also recognize the heterogeneity of the studies included as they assessed different clinical parameters, device brands, and settings. Nevertheless, this review contains information that can be useful for developing improved POCT devices designed not only considering patient, HCP and organization needs but moreover adapted for different settings. Furthermore, the use of our thematic analysis may be helpful in the implementation of POCT in different settings at different levels.

## Conclusions

This scoping review has described the facilitators and barriers of implementing a POCT device for cardiometabolic conditions. POCT allows quick turnaround of obtaining results and may facilitate decision-making and provision of care during the consultation generating patients' time-saving and satisfaction; however, the lack of clear clinical and regulatory guidelines, unsuitable infrastructure, and the increased workload for HCP hinder the sustainable utilization and implementation. The information provided in this review can be used to design better strategies to strengthen the facilitators and at the same time overcome the barriers to using POCT owing to proper planning involving stakeholders as well as end-user plus the provision of financial and physical resources. To achieve the WHO target of strengthening and orienting the health system to address the prevention and control of NCDs, it is needed that institutions and governments could enable access to diagnostics and technologies according to their priorities and resources. Thus, the implementation of POCT may benefit a wide population that today has no or limited access to cardiometabolic tests.

## Supplementary Information

**Additional file 1:** **Supplementary table 1. **Preferred Reporting Items for Systematic reviews and Meta-Analyses extension for ScopingReviews (PRISMA-ScR) Checklist.  

**Additional file 2.** 

**Additional file 3:** **Supplementarytable 3.** Characteristics of studies included in the scoping review.

## Data Availability

The authors declare that the data supporting the findings of this study are available within the article and its supplementary information files.

## Abbreviations

CFIR: Consolidated Framework for Implementation Research
CKD: Chronic kidney disease
CVD: Cardiovascular diseases
HCP: Health care professional
HIC: High income countries
HIV: Human Immunodeficiency Virus
HPV: Human Papillomavirus
LMICs: Low-and-middle income countries
POCT: Point-of-care testing
T2D: Type 2 diabetes
WHO: World Health Organization
