# Supplementary material for: Facilitators and barriers of the implementation of point-of-care devices for cardiometabolic diseases: a scoping review
Source: BMC Health Serv Res. 2023 Apr 28;23:412. doi: 10.1186/s12913-023-09419-2 (PMC10144879; doi:10.1186/s12913-023-09419-2)
Supplement: Supplementary file 2 — Additional file 2. [file 12913_2023_9419_MOESM2_ESM.pdf]

Ovid® | Wolters Kluwer | My Account | Support & Training | Ovid® Search Builder | Help | Feedback | Logoff

Search | Journals | Books | Multimedia | My Workspace | Links | What's New

**▼ Search History** (48) | View Saved

| # ▲ | Searches | Results | Type | Actions | Annotations |
|---|---|---|---|---|---|
| 1 | exp point-of-care systems/ | 21807 | Advanced | Display Results \| More | |
| 2 | point of care system.tw,kf. | 487 | Advanced | Display Results \| More | |
| 3 | point of care.tw,kf. | 72245 | Advanced | Display Results \| More | |
| 4 | point-of-care.tw,kf. | 72245 | Advanced | Display Results \| More | |
| 5 | point of care technology.tw,kf. | 452 | Advanced | Display Results \| More | |
| 6 | point of care testing.tw,kf. | 12265 | Advanced | Display Results \| More | |
| 7 | point-of-care testing.tw,kf. | 12265 | Advanced | Display Results \| More | |
| 8 | POC*.tw,kf. | 195893 | Advanced | Display Results \| More | |
| 9 | laboratory-independent.tw,kf. | 99 | Advanced | Display Results \| More | |
| 10 | rapid testing.tw,kf. | 4224 | Advanced | Display Results \| More | |
| 11 | near patient testing.tw,kf. | 743 | Advanced | Display Results \| More | |
| 12 | 1 or 2 or 3 or 4 or 5 or 6 or 7 or 8 or 9 or 10 or 11 | 261202 | Advanced | Display Results \| More | |
| 13 | primary care.tw,kf. | 339206 | Advanced | Display Results \| More | |
| 14 | primary health care.tw,kf. | 90392 | Advanced | Display Results \| More | |
| 15 | PHC.tw,kf. | 15593 | Advanced | Display Results \| More | |
| 16 | health care.tw,kf. | 1153337 | Advanced | Display Results \| More | |
| 17 | healthcare.tw,kf. | 811687 | Advanced | Display Results \| More | |
| 18 | general practice.tw,kf. | 94629 | Advanced | Display Results \| More | |
| 19 | health center.tw,kf. | 34003 | Advanced | Display Results \| More | |
| 20 | health centre.tw,kf. | 16975 | Advanced | Display Results \| More | |
| 21 | health service.tw,kf. | 136550 | Advanced | Display Results \| More | |
| 22 | social care.tw,kf. | 19271 | Advanced | Display Results \| More | |
| 23 | 13 or 14 or 15 or 16 or 17 or 18 or 19 or 20 or 21 or 22 | 2285488 | Advanced | Display Results \| More | |

Contract

| | | | | | | |
|---|---|---|---|---|---|---|
| ☐ | 24 | exp process assessment, health care/ | 3762084 | Advanced | Display Results \| More | |
| ☐ | 25 | process assessment, health care.tw,kf. | 634 | Advanced | Display Results \| More | |
| ☐ | 26 | implementation.tw,kf. | 787730 | Advanced | Display Results \| More | |
| ☐ | 27 | implementing.tw,kf. | 221637 | Advanced | Display Results \| More | |
| ☐ | 28 | exp delivery of health care/ | 5084606 | Advanced | Display Results \| More | |
| ☐ | 29 | delivery of health care.tw,kf. | 18515 | Advanced | Display Results \| More | |
| ☐ | 30 | delivery of healthcare.tw,kf. | 2836 | Advanced | Display Results \| More | |
| ☐ | 31 | health* delivery.tw,kf. | 18883 | Advanced | Display Results \| More | |
| ☐ | 32 | process evaluation.tw,kf. | 11642 | Advanced | Display Results \| More | |
| ☐ | 33 | quality improvement.tw,kf. | 126926 | Advanced | Display Results \| More | |
| ☐ | 34 | 24 or 25 or 26 or 27 or 28 or 29 or 30 or 31 or 32 or 33 | 8462372 | Advanced | Display Results \| More | |
| ☐ | 35 | facilita*.tw,kf. | 1475293 | Advanced | Display Results \| More | |
| ☐ | 36 | support*.tw,kf. | 4393519 | Advanced | Display Results \| More | |
| ☐ | 37 | assist*.tw,kf. | 1179898 | Advanced | Display Results \| More | |
| ☐ | 38 | enabl*.tw,kf. | 1249253 | Advanced | Display Results \| More | |
| ☐ | 39 | imped*.tw,kf. | 258945 | Advanced | Display Results \| More | |
| ☐ | 40 | obstruct*.tw,kf. | 819711 | Advanced | Display Results \| More | |
| ☐ | 41 | hinder*.tw,kf. | 167736 | Advanced | Display Results \| More | |
| ☐ | 42 | halt*.tw,kf. | 48768 | Advanced | Display Results \| More | |
| ☐ | 43 | prohibit*.tw,kf. | 66589 | Advanced | Display Results \| More | |
| ☐ | 44 | barrier*.tw,kf. | 878049 | Advanced | Display Results \| More | |
| ☐ | 45 | lesson*.tw,kf. | 189706 | Advanced | Display Results \| More | |
| ☐ | 46 | 35 or 36 or 37 or 38 or 39 or 40 or 41 or 42 or 43 or 44 or 45 | 9519132 | Advanced | Display Results \| More | |
| ☐ | 47 | 12 and 23 and 34 and 46 | 5986 | Advanced | Display Results \| More | |
| ☐ | 48 | remove duplicates from 47 | 4268 | Advanced | Display Results \| More | |

Save Remove Combine with: AND OR Deduplicate

Save All Edit Create RSS Create Auto-Alert View Saved Email All Search History Copy Search History Link Copy Search History Details
