# Supplementary material for: Facilitators and barriers of the implementation of point-of-care devices for cardiometabolic diseases: a scoping review
Source: BMC Health Serv Res. 2023 Apr 28;23:412. doi: 10.1186/s12913-023-09419-2 (PMC10144879; doi:10.1186/s12913-023-09419-2)
Supplement: Supplementary file 3 — Additional file 3: Supplementarytable 3. Characteristics of studies included in the scoping review. [file 12913_2023_9419_MOESM3_ESM.docx]

Supplementary table 3. Characteristics of studies included in the scoping review

| **First author, year, country** | **1. Study design 2. Study methods** | **Setting** | **POCT device under study** | **Parameter under study** | **Study aim** | **Main study findings** |
| --- | --- | --- | --- | --- | --- | --- |
| Al Hayek et al., 2021, Saudi Arabia ^[30]^ | 1. Cohort study 2. Quantitative | Hospital-based | Cobas B 101 (Roche) | HbA1c | To assess the level of satisfaction associated with the implementation of POCT for HbA1c testing in large tertiary care hospitals | The implementation of the POCT service reduced the number of test requested at laboratory level. The most common reasons for not performing HbA1c before the on-site service were difficulties in handling absence from school/work, inconvenience because of revisits, and additional travel costs. The mean HbA1c level has significantly improved after POCT implementation compared to the traditional HbA1c laboratory testing before POCT implementation. |
| Apple et al., 2006, United States ^[36]^ | 1. Retrospective audit design  2. Quantitative | Hospital cardiology service | Stratus CS STAT fluorometric system (Dade Behring) | Troponin I (cTnI) | To test whether substitution of routine, central laboratory testing of troponin with a POCT assay would result in an economic savings for patients presenting with symptoms suggestive of acute coronary syndrome | There was a decrease in time from blood draw to result to healthcare provider as well as a decrease trend in charge per patient admission ($4281 savings) following implementation of POC testing. Total charges per patient admission decreased by 25%. The mean length of stay also decreased.  Troponin I reagents charges to the laboratory were higher for the POC assay vs. the central lab assay. |
| Butler et al., 2021, United Kingdom ^[24]^ | 1. Implementation study 2. Qualitative | Outpatient clinics | Not specified | Lipid and HbA1c | To identify barriers and facilitators affecting implementation and engagement with POCT | Clinicians identified usability of the technology, positive impact on their patient’s experience and improved self-efficacy as drivers for successful implementation of POCT into their clinical practice. Issues with device functioning and the potential for a negative effect on the therapeutic relationship with their patients were identified as barriers. |
| Crocker et al. 2013, United States ^[26]^ | 1. Implementation study 2. Quantitative | Primary care practice | 1. Siemens DCA analyzer (Siemens Healthcare) 2. Abaxis Piccolo analyzer (Abaxis) | HbA1c Lipid and metabolic panel | To assess patient satisfaction with POCT for hemoglobin A1c, lipid panel and comprehensive metabolic panel in a primary care practice | On a scale of 1 (poor) to 4 (excellent), the mean of satisfaction score was 3.96. No difference in score among single POCT vs multiple test.  In 34 surveys a free text comment was included which was uniformly very positive. |
| De Vries et al., 2015, The Netherlands ^[18]^ | 1. Cross-sectional 2. Quantitative | General practice | Not specified | Hemoglobin, nitrites, and glucose | To evaluate safety aspects of POCT in general practitioners’ services | There is low adherence to quality control pre, during and post analysis. Results show there is not always attention for quality control measures (checking storage conditions, calibration, and maintenance). Universal hygienic measures (washing hands before taking a blood sample) are not always followed.  Refresher courses on the POC tests use are hardly organized. Only a few of the GPs contact the manufacturer of the device when a device failure occurs. |
| Dixon et al., 2021, United Kingdom ^[23]^ | 1. Cross-sectional 2. Qualitative | Out of hours primary care. | i-STAT (Abbott) | Cartridge CHEM 8 (sodium, potassium, calcium, glucose, creatinine, total carbon dioxide, anion gap, urea and hemoglobin). Cartridge CG4 (lactate and blood gases). | To explore clinicians’ perspectives on having POC tests available during out of hours home visits | Three themes were discussed: deciding whether to use POCT depending on patient or clinical situation and time/effort; supporting decision making and communication with patient, and gaining experience and confidence in using POC testing. |
| Eiff et al., 2011, Germany ^[22]^ | 1. Cross-sectional 2. Qualitative | Hospital-based | Not specified | Glucose | To describe constraints and resistance identified in the process of introduction of interconnected POC glucometers as part of the GLUMO study | Constraints were observed in the areas of enterprise culture or communication as well as the existing organization structure. Key players from laboratory, nursing and industry areas also identified various promoting and success factors to overcome the identified constraints: clear project planning, particularly process planning and time management; timely integration of all key players affected by the introduction of the innovation; transmission of necessary knowledge, possibly across all departments; and clear assignment of authorities as well as open communication structures. |
| Faulds et al., 2021, United States ^[27]^ | 1. Implementation study 2. Qualitative | Hospital based | Dexcom G6 (Dexcom) | Glucose | To describe the experience of implementing a CGM guideline to support POC glucose testing while on IV insulin in ICU patients with COVID-19 | Four major implementation themes were identified: accuracy, nursing ownership, workflow, and barriers and suggestions. Nurses’ perceived device accuracy and utility as exceptionally high. Solutions were devised to maximize facilitation and sustainability for nurses while maintaining patient safety |
| FitzGibbon et al., 2007, United Kingdom ^[21]^ | 1. Cross-sectional 2. Quantitative | Hospital-based |  | Cardiac marker testing, being troponin I the most relevant | To assess the value of POC technologies (views and opinions of healthcare professionals) for cardiac markers testing during the diagnosis of patients presenting chest pain | The main POCT users were clinical, nursing and laboratory staff. The implementation and accreditation should be overseen and regulated by the central laboratory. POCT should have the same quality of laboratory testing. Troponin I was the most important cardiac marker measured by POCT devices. |
| Geersing et al., 2010, The Netherlands ^[31]^ | 1. Prospective cohort study 2. Quantitative | Primary care | 1. Vidas D-dimer (Bio-Mérieux) 2. Pahfast D-dimer (Mitsubishi Cagaku Iatron)  3. Cardiac D-dimer (Roche Diagnostics)  4. Triage D-dimer (Biosite)  5. Clearview Simplify D-dimer (Inverness Medical) | D-dimer test | To compare accuracy of POCT D-dimer tests vs. leg-compression ultrasonography in patients with suspected deep vein thrombosis | All D-dimer tests showed negative predictive values higher than 98%. Sensitivity was high for all POC tests, with a range of 0.91 (Clearview Simplify) to 0.99 (Vidas). Specificity varied between 0.39 (Pathfast) and 0.64 (Clearview Simplify). The quantitative POC tests showed similar and high discriminative power for deep vein thrombosis. The ease of operation of the D-dimer tests and the risk of errors in the procedure were comparable among the 4 quantitative point-of-care D-dimer tests. |
| Hayward et al., 2020, United Kingdom ^[28]^ | 1. Implementation study 2. Mixed methods | Out-of-hours primary care | i-STAT (Abbott) | CHEM8 cartridge measures sodium, potassium, chloride, total carbon dioxide, anion gap, ionised calcium, glucose, urea, creatinine, haematocrit and haemoglobin. CG4 cartridge measures lactate, pH, PaO2 and PCO2, TCO2, bicarbonate, base excess, and oxygen saturation. | To describe the usage of tests across the period of implementation (home visits where tests were taken, clinical context of those tests, time taken to peform the test, and experienced problems) | i- STAT POC tests were infrequently used, with successful tests taken at just 47 contacts over time. The patients interviewed felt that testing had been beneficial for their care. Clinician interviews suggested barriers to POC tests, including practical challenges, concerns about time, doubt over whether they would improve clinical decision making and concern about increased medicolegal risk. Suggestions for improving adoption included sharing learning, adopting a whole team approach and developing protocols for usage. |
| Kost et al., 2006, Thailand and United States ^[20]^ | 1. Cross-sectional  2. Quantitative | Humanitarian crisis | Piccolo, i-STAT, Rapidpoint Coag, Orto-Clinical Diagnostics Blood Typing, Triage, Cardiac STATus | ABO blood group; cTnl, CK-MB, myoglobin; PT, aPTT, heparin management test, ACT, PT/INR; glucose, creatinine, Na+, K+, Cl-, Ca++, pH, PCO2, PO2, SO2, Hb, BUN, BNP; albumin, ALP, ALT, amylase, AST, HDL cholesterol, triglycerides, CK, TP, phosphorus, total bilirubin, direct bilirubin, TCO2, GGT, and uric acid. | To assess how POCT diagnostic testing at or near the site of patient care, can optimize diagnosis, triage, and patient monitoring during disasters | During disasters, limited availability and poor organization severely limited POCT use. The disasters broke some POCT and many victims seek health care.  Katrina demonstrated POCT value in disaster responses. Handheld POCT, airborne critical care testing, and disaster-specific mobile medical units should be available in small-world networks worldwide. |
| Laurance et al., 2010, Australia ^[16]^ | 1. Randomized controlled trial 2. Quantitative | General practice | Not specified |  | To compare satisfaction with POCT testing vs. laboratoy testing for three chronic conditions (diabetes, hyperlipidemia, and antocoagulant therapy) | Intervened patients reported they were satisfied with POC testing. Compared to the control group, the intervention group had a higher level of agreement with statements relating to their satisfaction with the collection process and confidence in the process.  They also viewed POC testing as strengthening their relationship with their practitioners and motivational in terms of better managing their condition. |
| Marley et al., 2015, Australia ^[19]^ | 1. Cross-sectional 2. Quantitative | Primary healthcare practices | DCA 2000+ Analyzer (Siemens, Bayer) | HbA1c | To assess the accuracy of POC HbA1c testing in diagnosis diabetes comparing capillary with venous blood samples | Concordance between POC and laboratory results was good, and the mean difference was small.  POC HbA1c measurements ≥6.5% had a specificity of 98.2% and sensitivity of 73.7% for laboratory measurements ≥6.5%. The POC equivalence value for screening for diabetes or a high risk of developing diabetes was ≥5.7% (sensitivity 91%, specificity 76.7%, for laboratory measurements ≥6.0%).  Staff reported difficulty in maintaining formal accreditation. |
| Mash et al., 2016, South Africa ^[34]^ | 1. Quasi-experimental study 2. Mixed methods | Primary community health care centers | DCA Vantage Analyzer (Siemens) | HbA1c | To evaluate the costs and consequences of introducing POC testing for HbA1c in T2DM patients | In the control group, 8.3% additional patients were referred for counselling compared with 1.7% fewer in the intervention group. POC testing was feasible, easy to integrate into the organisation of care, resulted in more immediate feedback to patients (p<0.001) and patients appeared more satisfied. POC testing did not improve test coverage, treatment intensification, counselling or glycaemic control.  There was an incremental cost of ZAR 2110 per 100 tests.  Compliance with quality control was poor, although control tests showed good reliability. |
| Norman et al., 2022, New Zealand ^[37]^ | 1. Observational pilot evaluation 2. Quantitative | General practice | i-STAT c-TnI (Abbott) | Cardiac troponin I | To determinet the proportion of patients managed using the low-risk pathway without transfer to hospital and without 30-day major adverse cardiac event | A total of 180 patients were assessed by the new pathway. Over 60% of patients were identified as low-risk for AMI and managed in rural general practice without 30-day MACE. Adherence to the low-risk pathway was 95.5%.  A structured pathway is feasible to implement, and initial results suggest it is possible to safely and markedly reduce hospital transfer of low-risk patients presenting to rural general practice with suspected cardiac chest pain, negating the requirement for often lengthy transfers to rural or base hospitals for further assessment and serial troponin testing. |
| Peirce et al., 2015, United Kingdom ^[35]^ | 1. Case studies 2. Qualitative | National health services | Not specified | INR (coagulometer) C-reactive protein | To explain non-adoption and alleged under-adoption of a number of technologies in the UK NHS | Despite the positive evidence of the two devices, they were not widely used. CRP testing was not attractive due to a weak utility identity coupled with high financial and pragmatic requirements. POCT for anticoagulation was adopted because its requirement for widespread organisational change enabled the redesign of dysfunctional services, but self-testing was not encouraged. |
| Shephard et al., 2012, Australia ^[25]^ | 1. Implementation study 2. Mixed methods | Primary healthcare centers | i-STAT 300 analyzer (Abbot Diagnostics) | The Chem8+ cartridge measures electrolytes, total CO2, urea, creatinine, glucose, ionised calcium and haemoglobin.  A different cartridge measures INR for patients on warfarin therapy. The CG4+ cartridge measures blood gases and lactate. The Troponin cartridge measures cardiac troponin I). | To improve pathology services in remote health centres through the implementation of a quality managed POCT service | Health professional staff (n =164) were trained to perform i-STAT POCT during the first year of the program. A total of 2290 POCT tests were performed on the i-STAT. The volume of testing consistently increased across the year. Stakeholder satisfaction with the device was high, with an improvement in satisfaction levels with pathology service provision.  Remote health professionals reported a significantly high level of satisfaction with point-of-care testing for servicing acutely ill patients. |
| Wake et al., 2022, Australia ^[29]^ | 1. Implementation study 2. Mixed methods | Tertiary hospital | ROTEM (Werfen) | Prothrombin time (PT) and activated partial thromboplastin time (aPTT) | To evaluate the implementation data to identify factors which helped and hindered this new practice | The were no good adherence to the trauma activation protocol. Patients who met the trauma activation criteria increased accross the time of implementation, 63% in the sustainability phase. Qualitative data suggest that a more tailored approach to intervention implementation would assist with adoption. |
| Wells et al., 2017, New Zealand ^[32]^ | 1. Pragmatic cluster randomized controlled trial 2. Mixed methods | General practice | Cobas B 101 (Roche Diagnostics International) | Total cholesterol/high density lipoprotein (TC/HDL) | To determine the proportion of completed CVD risk assessment conducted for eligible patients | Having a POC testing device within the practice made no difference to the completion of CVD risk assessments.  There were major external influences that affected the trial: including a national performance target for CVD risk assessment and changes to CVD guidelines. The POC device was viewed as an additional tool rather than as an opportunity to review practice workflow and leverage the immediate test results for patient education and CVD risk management discussions. |
